# Ae Fond Fareweel^1^

**DOI:** 10.1093/plphys/kiab467

**Published:** 2021-12-04

**Authors:** Mike Blatt

**Affiliations:** Laboratory of Plant Physiology and Biophysics, Bower Building, University of Glasgow, Glasgow G12 8QQ, UK

Sitting down to write this editorial, I recognize my ambivalence. These past 9 years, during which I have served as Editor-in-Chief of *Plant Physiology*, mark a period of transitions, successes, and the occasional disappointment, but most of all of a sense of satisfaction, both personal and professional. The American Society of Plant Biologists for their part must agree, as they saw fit to reappoint me to the post twice. Their trust in me has been deeply gratifying. Yet, while I see that there is still much to do going forward, I also recognize that it is time for new blood at the helm. So, my ambivalence.

The question most often asked of me is whether the role of Editor-in-Chief has not been a huge task. Of course, it has; but it comes with equal rewards. I take immense pride in the way that *Plant Physiology* has grown; in the dedicated, face-to-face meetings of the editorial board that I introduced in 2014 and how we have come together as a working community; in the knowledge that my support has helped to guide the board through a number of challenges, not least in developing policies that have extended journal coverage, doubled its annual citation count—for 2021 expected to exceed 100,000 citations—and helped raise its impact factor from 6.55 to 8.34. Indeed, *Plant Physiology* remains by far the single, most highly cited international journal in the plant sciences.

Make no mistake, the success of *Plant Physiology* would not have been possible without this community: the editors who rightly can take ownership of the journal and its achievements, and the staff, past and present, who have fostered a supportive familial atmosphere within the Society. My deepest thanks to you, not only for your unstinting efforts, but also for your friendship. Working with the journal these past 9 years, I have come to know many with whom I might not have crossed paths otherwise, and this has been the greatest pleasure for me.

There is much I might relate from my experiences and no single theme that can encapsulate them all. So I highlight here a few points only. Foremost on my mind are the changes to scientific publishing. These have been breathtaking, in many ways benefitting authors and readers, and occasionally disturbing in their implications. While I recognized from the start that scientific publishing was moving toward online staging as the primary means of dissemination, I could not have envisaged its pace, nor the influence it has had in expanding author-pay and open-access platforms, the explosion of online (and sometimes predatory) journal titles, and the evolving “S plan” for public access to scientific output.

Even as they continue to develop, these changes are completely transforming the landscape of academic publishing, not to mention the research that it showcases, and they present special challenges for the survival of society journals. There is a cliché about unintended consequences (in military terms, “collateral damage”); I often wonder whether the advocates of the S plan, themselves, were caught unawares by its consequences for scientists, their access to publication as authors, not just as readers and, looking forward, its potential for further polarizing research globally.^2^ For researchers in many countries where a laboratory may operate with a total annual budget around US$5,000, often much less, these transformations do no more than swap paywalls for publishwalls, article processing charges that are unaffordable and effectively “lock” researchers out of opportunities to gain visibility.

The digital transformation has generated other challenges that will continue to demand attention. Among these, not least are the growth of social media and so-called post-publication peer review (PPPR). Forgive me if I risk a few generalizations (as Alexandre Dumas once noted, “All generalizations are dangerous, even this one”). A great strength of social media is the way it has encouraged public access to science as well as accelerating its dissemination within the research community. It is a worry, however, that social media endorses, if unwittingly, a deep-seated impatience that does not always sit well with considered scientific thought. I noted previously that “publication is only the beginning of scientific debate” (Blatt, 2016); informed discussion is vital, whether online or in person, but it retains value only to the extent that it remains open (non-anonymous), courteous, and measured. These are a minimum for any constructive exchange; they are all the more essential in the public setting of online discourse and so important to avoid misinformation that otherwise becomes astoundingly difficult to counter (Bergstrom and West, 2021).

Which brings me to the anonymous airings of PPPR. Here I see great danger. Early on, I shared my views in a series of editorials calling out the public forum of PubPeer that purports to host PPPR (Blatt, 2015, 2016; da Silva and Blatt, 2016). The first of these editorials sparked an extensive (and largely anonymous!) argument on PubPeer itself that spilled over onto PubMed Commons but was shunned by Retraction Watch. At the heart of the issue is whether anonymity has a place in scientific discourse. I maintain that it does not.

For me, it remains a deep concern that open debate is not lost, both for science today and for the future. Bear in mind that the process of scientific critique differs from that of policing. Websites such as PubPeer fail to make this distinction; they make the case for anonymity in scientific discourse by conflating the objectives of debate with those of research integrity. For sure, there is a legitimate place for anonymity in protecting the whistleblower, but not in scientific debate.

Setting aside policing, anonymity in PPPR creates an atmosphere that undermines informed discussion and, more broadly, the public perception of science. I noted that anonymity is intimidating and confrontational by its very nature, it entrenches inequality in every exchange, and the price often enough is an absence of worthwhile discussion. As one commenter in the ensuing PubPeer threads^3^ noted, scientific debate “centers around the exchange of ideas … [and] is based on the pillars of knowledge, expertise and transparency. The whole thing collapses when any one of these three fails. … it’s a ‘no-brainer’ that anonymity on social media (what an excellent oxymoron it is, too, in this context) breaks the social contract of an open discussion.”

As scientists, we are trained to question ideas and the evidence that supports them, to debate and assess alternative interpretations, and to test predictions that arise from these deliberations. Progress in science is a massively cooperative undertaking and its foundations are built on communication. Thus, “it is a warped worldview, indeed, in which scientists are so fearful of engaging that they never challenge others’ research and ideas openly, whether online or in publication” (Blatt, 2015, 2016).

As for scientific integrity, there can be no doubt that digital platforms have expanded an awareness of data mishandling, misinformation, and occasionally fraud within the scientific publications. This is not to say that we are seeing an explosion of fraudulent research. Over 30 years of analysis by the Rockefeller University Press and by the NIH has shown that the rate of deliberately fraudulent papers remains virtually unchanged, present in a fraction of a percent only of research publications (Blatt, 2016). Nonetheless, digital media certainly offers new avenues for data manipulation, whether out of ignorance or otherwise, and has necessitated a proportionate response from publishers.

We introduced routine checks of data presented in all manuscripts accepted by *Plant Physiology*, beginning in January 2018. These checks have supported the findings of the Rockefeller University Press and the NIH, and they have unquestionably improved the quality of research that the journal publishes. If I have a disappointment here, it lies in the difficulty to convince, beyond this Society, in favor of a coordinated, plant science-wide approach to scientific fraud. Nonetheless, I am pleased that the Committee on Publication Ethics (COPE) now recognizes the benefits of sharing relevant information between journals.^4^

One unalloyed success, also facilitated by the digital platform of the Society, is the Associate Features Editor program. *Plant Physiology* introduced the program in 2018 alongside the *Plant Cell* to bring promising early-career scientists onto the editorial board and engage their expertise in assessing and writing about research published in the journal. It has been a pleasure to watch these scientists bring their passion for science to the journal and to see their News and Views articles on some of the most exciting advances we publish. Their contributions continue to expand the journal content and, equally, they gain professionally in experience and exposure. I have shared the task of handling and editing the Associate Features Editor submissions with staff members Mary Williams and Ash Wolf, and we take great pride in their progress. Indeed, the majority of the early-career scientists we worked with in the first 2 years of the program have since secured permanent academic and research posts. I like to think that, in our small way, we have helped to support their successes.

I am equally proud of the manner in which the scope of *Plant Physiology* has expanded over the past decade without losing its core themes, including those of biochemistry, molecular biology, and hormonal physiology. The generic approach to both remit and formats that I brought to the journal has helped to blur research boundaries, support and encourage submissions that integrate methods and straddle disciplines; it has facilitated rapid and focused publication, for example, with the formats of RESEARCH REPORTs and LETTERs that attract much reduced and no author charges, respectively; and it has granted the journal a flexibility that opens the doors to new developments. Among the latter, both ecophysiology and synthetic biology are growing concerns that have found a home with the journal over the past decade and will no doubt grow further over the next.

Most of all, I take pride in the way that the Society and *Plant Physiology* have built an environment that serves the plant sciences with a publication of the highest standards while nurturing and supporting authors, both those who aspire to publish with the journal and those who do. It has been a great pleasure for me to contribute in this construct. Looking forward now, the immediate tasks ahead will be in establishing with the new partner of the Society, Oxford University Press, a seamless operation that fosters and enhances the standards of *Plant Physiology* while ensuring this legacy of support for the research community and for authors. These are tasks that I am certain the incoming Editor-in-Chief Yunde Zhao is ready to take on, and I wish him the very best of success as the journal enters its second century.

## Footnotes

2 See doi.org/10.1038/d41586-021-00883-6; chemistryworld.com/news/blow-to-open-access-plan-s-as-european-research-council-withdraws/4012244.article; the-scientist.com/news-opinion/as-plan-s-takes-effect-some-anticipate-inequitable-outcomes-69058.

3
pubpeer.com/publications/209CA2DF493322A5B5470F3-B8EEDA0

4
publicationethics.org
